# Effects of Asian Dust Particles on the Early-Stage Antigen-Induced Immune Response of Asthma in NC/Nga Mice

**DOI:** 10.3390/ijerph13111144

**Published:** 2016-11-16

**Authors:** Jun Kurai, Masanari Watanabe, Hiroyuki Sano, Degejirihu Hantan, Yuji Tohda, Eiji Shimizu

**Affiliations:** 1 Department of Respiratory Medicine and Rheumatology, Faculty of Medicine, Tottori University, 36-1 Nishi-cho, Yonago 683-8504, Japan; junkurajun@gmail.com (J.K.); degujirefu@med.tottori-u.ac.jp (D.H.); eiji@med.tottori-u.ac.jp (E.S.); 2 Department of Respiratory Medicine and Allergology, Kinki University, 377-2 Ohnohigashi, Osakasayama 589-0014, Japan; hsano@med.kindai.ac.jp (H.S.); tohda@med.kindai.ac.jp (Y.T.)

**Keywords:** airway inflammation, asthma, Asian dust, leukotrienes, NC/Nga mouse model

## Abstract

Asian dust (AD) can aggravate airway inflammation in asthma, but the association between AD and the development of asthma remains unclear. This study aimed to investigate the effects of AD on the early stage of antigen sensitization using a mouse model of asthma, as well as the role of leukotrienes (LTs) in antigen-induced airway inflammation potentiated by AD particles. NC/Nga mice were co-sensitized by intranasal instillation of AD particles and/or *Dermatophagoides farinae* (Df) for five consecutive days. Df-sensitized mice were stimulated with an intranasal Df challenge at seven days. Mice were treated with the type 1 cysteinyl LT (CysLT_1_) receptor antagonist orally 4 h before and 1 h after the allergen challenge. At 24 h post-challenge, the differential leukocyte count, inflammatory cytokines, and LTs in bronchoalveolar lavage fluid were assessed, and airway inflammation was evaluated histopathologically. AD augmented neutrophilic and eosinophilic airway inflammation with increased CysLTs and dihydroxy-LT in a mouse model of asthma. The CysLT_1_ receptor antagonist was shown to attenuate both neutrophilic and eosinophilic airway inflammation augmented by AD. Therefore, exposure to AD may be associated with the development of asthma and LTs may play important roles in airway inflammation augmented by AD.

## 1. Introduction

The health burden of airborne particulate matter (PM) is an important environmental health concern, and a number of studies have demonstrated that airborne PM exposure can increase respiratory and cardiovascular morbidity and mortality [1,2]. Desert sand dust particles are an important component of airborne PM and can have great effects on health over large distances because they can travel far from their source [3,4]. Asian dust (AD) emissions originating from East Asian deserts in Mongolia, Northern China, and Kazakhstan produce the second largest volume of dust emissions worldwide, and account for approximately 20% of the global total [5]. AD emissions most frequently occur from February to May, although data suggests that they may last until late autumn [5,6,7]. AD particles are continuously carried to Japan, especially from February to May [6,7]. Numerous epidemiological studies have found that AD emissions have effects on respiratory and cardiovascular morbidity and mortality [3,8]. Similarly, AD emissions are associated with asthma exacerbation [9,10,11,12,13,14,15]. The role of exposure to airborne PM in the development of asthma remains unclear. Recently, several studies have found that exposure to airborne PM early in life may contribute to the development of asthma [16,17]. However, no previous study has investigated the association between long-term exposure to AD and the development of asthma.

Leukotrienes (LTs) are a family of eicosanoid inflammatory mediators that are produced from the metabolism of arachidonic acid via the 5-lipoxygenase pathway in various cells (including eosinophils, mast cells, and alveolar macrophages) and in response to airway stimuli [18]. There are two classes of LTs: the cysteinyl LTs (CysLTs), such as LTC_4_, LTD_4_, and LTE_4_; and the dihydroxy-LTs (LTB_4_) [18]. LTs play important roles in asthma, with each class acting through two structurally divergent G protein–coupled receptors: the type 1 and type 2 CysLT receptors (CysLT_1_ and CysLT_2_ receptors, respectively) [19,20]. CysLTs can induce strong bronchoconstriction, eosinophilic inflammation, and mucus secretion [19]. Therefore, the LT receptor antagonist is commonly used in the management of asthma.

In contrast to bronchoconstriction mediators (i.e., the CysLTs), LTB_4_ is thought to be a pro-inflammatory mediator that is responsible for the recruitment, activation, and survival of leukocytes, including neutrophils and eosinophils [21,22,23,24]. Several studies have reported that airborne PM is associated with increased in vivo concentrations of CysLTs [25,26]. In humans, Rabinovitch et al. found that the serum concentrations of LTE_4_ in children with asthma were significantly associated with the levels of PM in their living areas [27]. However, the roles of LTB_4_ in asthma-associated airway inflammation augmented by PM are still undefined.

The present study aimed to evaluate the effects of AD on early-stage antigen sensitization using a mouse model of asthma. Furthermore, the concentrations of CysLTs and LTB_4_ in bronchoalveolar lavage fluid (BALF) were measured to evaluate their roles in antigen-induced airway inflammation potentiated by AD particles. This study also investigated whether a CysLT_1_ receptor antagonist could abolish these initial immune responses augmented by AD particles.

## 2. Materials and Methods

### 2.1. Animals

Specific pathogen-free seven-week-old male NC/Nga mice were purchased from Japan SLC Inc. (Hamamatsu, Japan) and acclimatized for seven days before the start of the study. Animals were kept in a vivarium at a constant temperature of 22 °C and illumination in 12 h light/dark cycles. Animals were fed standard animal chow daily and had ad libitum access to drinking water. The experimental protocols were approved by our institutional Animal Care and Use Committee (Faculty of Medicine, Tottori University; protocol number 13-Y-5).

### 2.2. Preparation of AD Particles

Airborne particles were collected in Tottori on AD days during 18–22 March 2013, using a high-volume air sampler (HV-1000R; Shibata Co., Tokyo, Japan) that was fixed on the rooftop of a building. AD particles were separated according to their aerodynamic diameters using five filters (<1.1 μm, 1.1–2.0 μm, 2.0–3.3 μm, 3.3–7.0 μm, and >7.0 μm), and each filter was dried in a desiccator and weighed before and after sampling. The size distribution of AD particles is primarily 3.3–7.0 μm, and the AD particles for the present study were selected from the 3.3–7.0 μm filter. The AD particles were sterilized at 121 °C for 30 min in an autoclave (Tomy SX-300; Tomy Co., Tokyo, Japan) and stored in a freezer at −20 °C to prevent the growth of bacteria and fungi. The AD particles were suspended in normal saline (NS) before being administered to the mice.

### 2.3. Experimental Protocol

NC/Nga mice were sensitized to *Dermatophagoides farina* (DF; Greer Laboratories Inc., Lenoir, NC, USA), as previously described [28]. After a seven-day acclimatization period, the mice were randomly divided into eight groups (*n* = 8 per group). For sensitization, the mice were anesthetized using isoflurane inhalation and underwent intranasal instillation of Df crude extract in NS (50 μg/25 μL) for five consecutive days (days 0–4). Df-sensitized mice were challenged intranasally using Df at seven days after the last Df sensitization (day 11) and sacrificed 24 h after the Df challenge. In the control group, NS was administered instead of Df sensitization.

**Figure 1:**
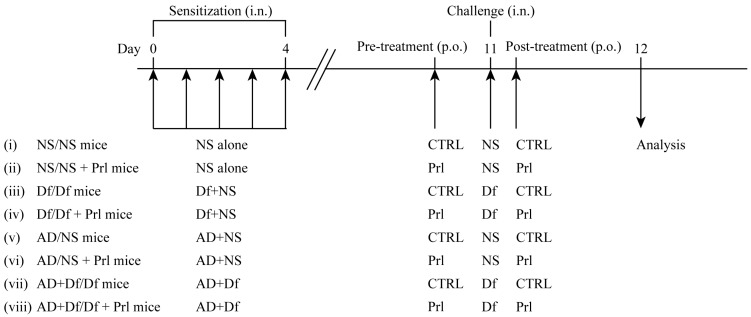
Experimental protocol. NC/Nga mice were sensitized using intranasal (i.n.) installation of a mixture (Asian dust (AD) particles and/or *Dermatophagoides farina* (Df), or CJ-1 soil and/or Df) for five consecutive days (days 0–4). Pranlukast (Prl)-treated mice received Prl orally (25 mg/kg/day) at 4 h before the allergen challenge and at 1 h after the challenge on day 11. At seven days after the last allergen sensitization, the mice were challenged using the allergen, which was followed by the collection of bronchoalveolar lavage fluid, lung tissue, and serum. Mice were divided into eight groups: normal saline (NS)/NS, NS/NS + Prl, Df/Df, Df/Df + Prl, AD/NS, AD/NS + Prl, AD + Df/Df, and AD + Df/Df + Prl.

To observe the effects of a CysLT_1_ receptor antagonist (Pranlukast (Prl); Ono Pharmaceutical Co., Ltd., Osaka, Japan) on airway inflammation induced using AD particles (0.1 mg/25 μL of NS), mice were co-sensitized using intranasal instillation of AD particles and/or Df for five consecutive days (days 0–4) (Figure 1). Prl was dissolved in 0.5% carboxymethylcellulose sodium (CMC) and was administered using a gastric tube. Prl-treated mice received Prl orally (25 mg/kg/day) at 4 h before the allergen challenge and at 1 h after the challenge on day 11. In the control group, 0.5% CMC was administered orally in the same manner. The eight groups were as follows: (i) NS/NS mice, which were sensitized and challenged using NS; (ii) NS/NS + Prl mice, which were sensitized and challenged using NS, and treated with Prl; (iii) Df/Df mice, which were sensitized and challenged using Df; (iv) Df/Df + Prl mice, which were sensitized and challenged using Df, and treated with Prl; (v) AD/NS mice, which were sensitized using AD particles and challenged using NS; (vi) AD/NS + Prl mice, which were sensitized using AD particles, challenged using NS, and treated using Prl; (vii) AD + Df/Df mice, which were co-sensitized using Df and AD particles, and challenged using Df; and (viii) AD + Df/Df + Prl mice, which were co-sensitized using Df and AD particles, challenged using Df, and treated with Prl.

### 2.4. BALF Procedure

After the mice were anesthetized using isoflurane, their tracheas were cannulated. BALF was subsequently obtained after five instillations of NS (1.0 mL) into the lungs, along with gentle handling to maximize BALF recovery. BALF from each mouse was centrifuged at 300× *g* for 5 min at 4 °C. The cell pellet was used for cell counting, and the supernatant was used for cytokine analysis. Total cells diluted in Turk’s fluid were counted using a hemocytometer. The differential leukocyte count was performed using microscopic evaluation and quantitative analysis of methanol-fixed cytospin preparations stained using Diff Quick (Thermo Fisher Scientific, Inc., Pittsburgh, PA, USA).

### 2.5. Histological Examination

Mice were euthanized using a pentobarbital injection. Lungs were inflation-fixed at 25 cm of water pressure with 10% formalin for 5 min and immersed in the same fixative. Tissues were fixed for 24 h at 4 °C and processed using standard methods for paraffin-embedded blocks. Fixed lung tissues were embedded in paraffin and sectioned, and each section was stained using hematoxylin and eosin (H&E).

### 2.6. Quantitative Determination of Cytokine, Chemokine, and LT Levels

Interferon (IFN)-γ, interleukin (IL)-13, IL-5, IL-6, keratinocyte-derived chemokine (KC/CXCL1), and macrophage inflammatory protein (MIP)-2 (KC/CXCL1 and MIP-2/CXCL2 are murine homologues of human IL-8) levels in BALF were measured using enzyme immunoassay (EIA) kits (R&D Systems Europe, Abingdon, UK). Granulocyte-macrophage colony stimulating factor (GM-CSF) levels in serum and BALF were measured using EIA kits (R&D Systems Europe), as were CysLTs and LTB_4_ levels (Cayman Chemical, Ann Arbor, MI, USA). BALF dilutions were 1:5 for IL-13, IL-5, IL-6, KC/CXCL1, and MIP-2/CXCL2. BALF dilutions were 1:2 for CysLTs and LTB_4_. BALF was undiluted for IFN-γ. Serum was undiluted for GM-CSF. All EIA assays were performed according to the manufacturer’s instructions.

### 2.7. Statistical Analysis

Data are expressed as the mean ± standard deviation. Comparisons between the groups were made using a one-way analysis of variance. Calculations were performed using GraphPad Prism (ver. 5.02; GraphPad Software, San Diego, CA, USA). A *p*-value of <0.05 was considered statistically significant.

## 3. Results

### 3.1. Cell Counts in BALF

**Figure 2:**
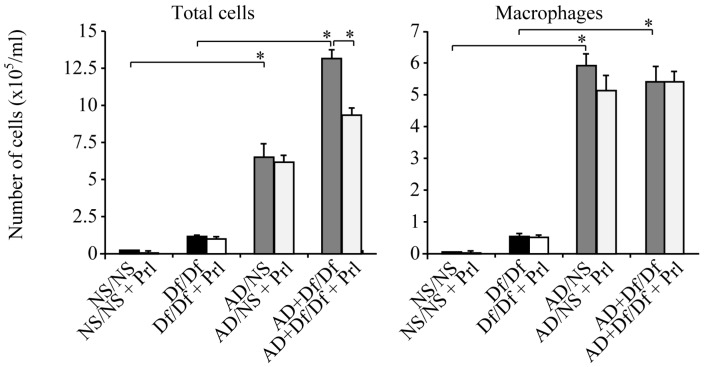

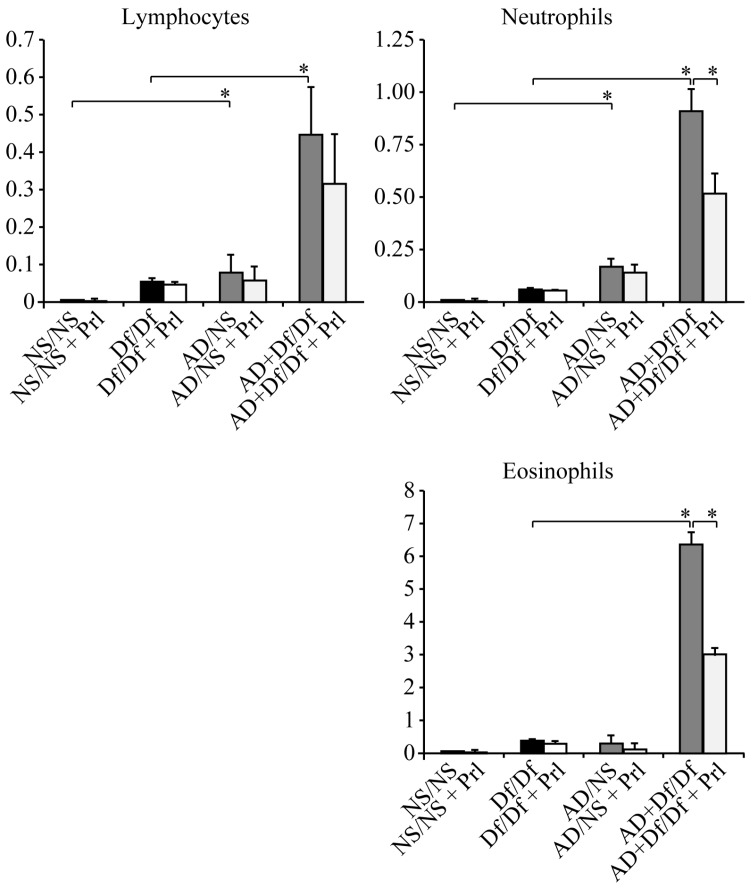
Total and differential leukocyte counts in bronchoalveolar lavage fluid (BALF). The cell counts in BALF were obtained 24 h after the allergen challenge on day 11. The differential leukocyte counts included macrophages, lymphocytes, neutrophils, and eosinophils. Total cell counts in Asian dust (AD) + *Dermatophagoides farina* (Df)/Df + Pranlukast (Prl) mice were significantly decreased, as compared with AD + Df/Df mice. Data are expressed as the mean ± standard deviation, with eight mice per group. * *p* < 0.05.

Df/Df mice had a significantly increased BALF total cell count compared with control NS/NS mice (*p* < 0.05). Mice co-sensitized using AD particles and Df (AD + Df/Df mice) had greater enhancement of airway inflammation compared with Df/Df mice, and the total cell count was significantly increased (11.2-fold) in AD + Df/Df mice (Figure 2). The increased cell count was consistent for macrophages, eosinophils, lymphocytes, and neutrophils in AD + Df/Df mice, as compared with Df/Df mice (Figure 2). The increased neutrophil count was most noticeable in the differential leukocyte count, with a significant (20.2-fold) elevation in AD + Df/Df mice, as compared with Df/Df mice. Prl treatment alone had no effect on cell counts in NS/NS and Df/Df control mice.

Prl-treated mice co-sensitized using AD particles and Df (AD + Df/Df + Prl mice) exhibited a significantly decreased BALF total cell count, as compared with AD + Df/Df mice (*p* < 0.05; Figure 2). The total cell count was significantly decreased by 29.6% in AD + Df/Df + Prl mice. Prl treatment did not affect the total cell counts in AD/NS mice. The decreased cell counts were significant and consistent for eosinophils (52.4%) and neutrophils (43.1%) in AD + Df/Df + Prl mice, as compared with AD + Df/Df mice (*p* < 0.05; Figure 2).

### 3.2. Cytokine Profile of BALF

**Figure 3:**
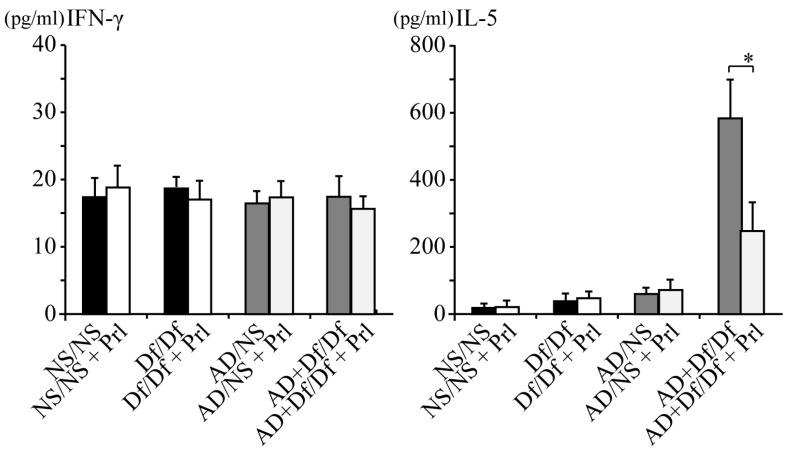

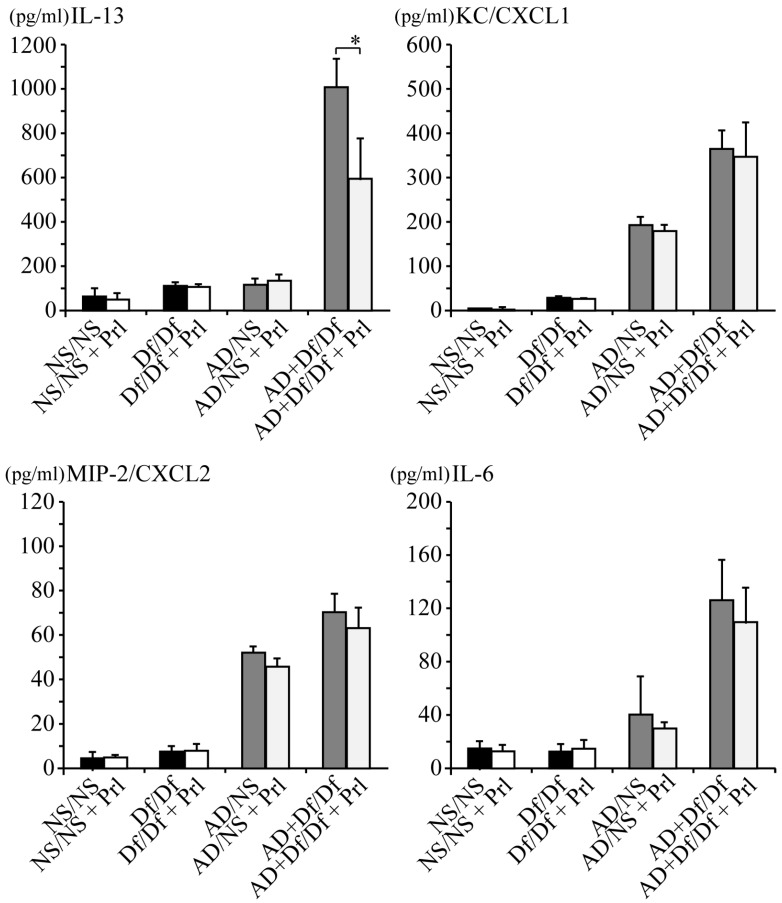
Cytokine and chemokine levels in bronchoalveolar lavage fluid (BALF). BALF cytokine and chemokine expression profiles were analyzed using enzyme immunoassays for interferon (IFN)-γ, interleukin (IL)-5, IL-13, keratinocyte-derived chemokine (KC/CXCL1), macrophage inflammatory protein-2 (MIP-2/CXCL2), and IL-6. Data for each group are expressed as the mean ± standard deviation, with six mice per group. * *p* < 0.05.

Cytokine levels in BALF were measured to investigate the mechanisms through which Prl attenuates the allergic airway response induced by AD particles in the mouse model of Df-induced asthma. In parallel with the inflammatory cell recruitment in BALF, AD particles induced the production of several cytokines that are important in the development of asthma. The levels of T-helper 2 (Th2) cytokines (IL-5 and IL-13) in BALF from AD + Df/Df mice were significantly decreased by Prl treatment (*p* < 0.05; Figure 3), while the Th1 cytokine (IFN-γ) and inflammatory cytokines’ (IL-6, MIP-2/CXCL2, and KC/CXCL1) levels were not significantly decreased after Prl treatment.

### 3.3. Histopathological Changes in the Lung

**Figure 4:**
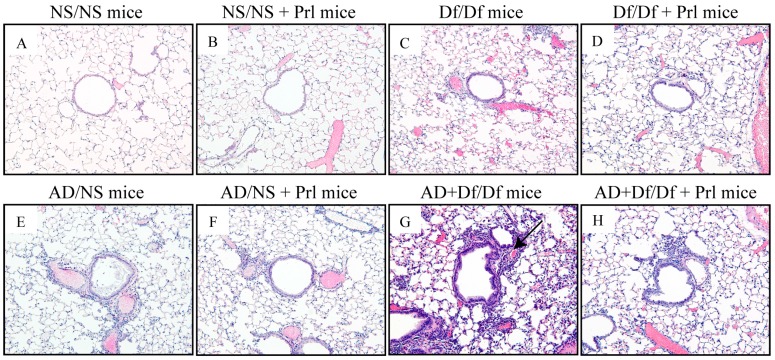
Effects of Pranlukast (Prl) treatment on histopathological changes in the lungs. Light photomicrographs of representative lung sections were stained using hematoxylin and eosin (magnification: ×200). Representative light photomicrographs of normal saline (NS)/NS mice (**A**); NS/NS + Prl mice (**B**); *Dermatophagoides farina* (Df)/Df mice (**C**); Df/Df + Prl mice (**D**); Asian dust (AD)/NS mice (**E**); AD/NS + Prl mice (**F**); AD + Df/Df mice (**G**); and AD + Df/Df + Prl mice (**H**). Arrow heads show peribronchiolar and perivascular mixed inflammatory cell infiltration in AD + Df/Df mice.

Lung specimens were evaluated using H&E staining to determine the histopathological effects of Prl treatment on the airway. Df/Df mice had greater peribronchiolar and perivascular inflammatory cell infiltration, as compared with control NS/NS mice. Greater airway inflammation was also apparent in AD + Df/Df mice, as compared with Df/Df mice (Figure 4). AD + Df/Df + Prl mice exhibited relatively weak inflammatory responses, as compared with AD + Df/Df mice. These histopathological findings were consistent with the BALF analyses that revealed significant decreases in eosinophil and neutrophil counts after Prl treatment.

### 3.4. Measurements of CysLTs and LTB_4_ in BALF

**Figure 5:**
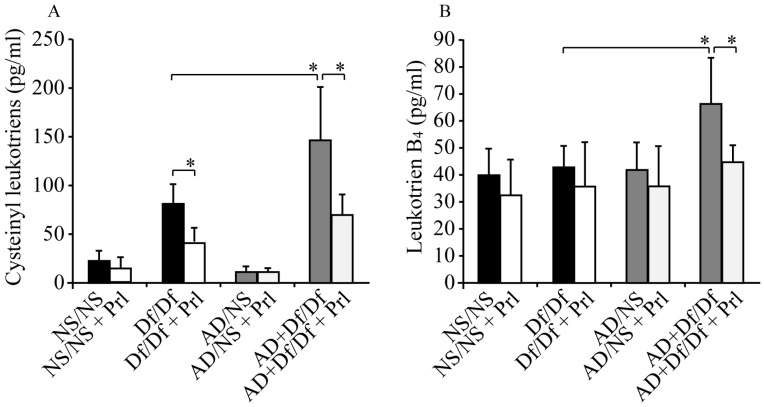
Effects of Pranlukast (Prl) treatment on cysteinyl leukotrienes (CysLTs) and dihydroxy-LT (LTB_4_) levels in bronchoalveolar lavage fluid (BALF). BALF LT production was measured using enzyme immunoassays for CysLTs (**A**) and LTB_4_ (**B**). Data for each group are expressed as the mean ± standard deviation, with six mice per group. * *p* < 0.05.

AD + Df/Df and Df/Df mice exhibited higher CysLT levels in BALF as compared with AD/NS and NS/NS mice. Furthermore, AD + Df/Df mice exhibited higher levels of CysLT compared with Df/Df mice. The increased CysLTs levels in AD + Df/Df and Df/Df mice were significantly reduced by Prl treatment. CysLTs levels in AD + Df/Df + Prl mice were 52.2% lower than those in Df/Df + Prl mice (*p* < 0.05; Figure 5A). Similarly, AD + Df/Df mice exhibited significantly higher LTB_4_ levels as compared with Df/Df mice (*p* < 0.05; Figure 5B). Prl treatment also significantly decreased LTB_4_ levels in BALF (*p* < 0.05; Figure 5B).

### 3.5. Measurement of GM-CSF in Serum and BALF

**Figure 6:**
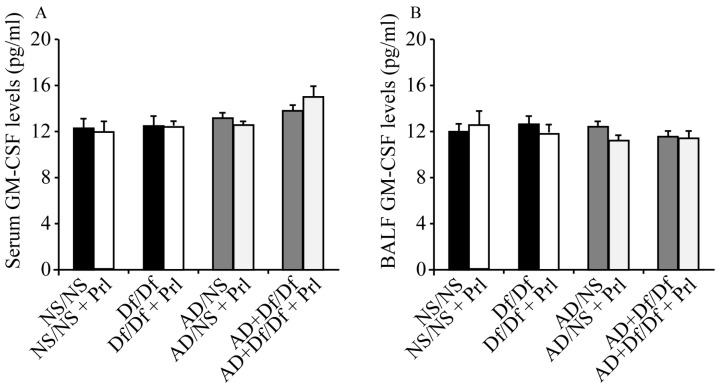
Granulocyte-macrophage colony stimulating factor (GM-CSF) levels in serum and bronchoalveolar lavage fluid (BALF). GM-CSF levels in serum (**A**) and BALF (**B**) were analyzed using enzyme immunoassays. Data for each group are expressed as the mean ± standard deviation, with six mice per group.

GM-CSF levels in serum and BALF were measured to investigate the mechanisms underlying the decreased BALF values for neutrophils and LTB_4_ following treatment with Prl. GM-CSF levels were not significantly decreased after Prl treatment (Figure 6).

## 4. Discussion

The present study revealed that AD particles can augment airway inflammation in a Df-sensitized mouse model of asthma. The data also revealed significant increases in the number of total cells, neutrophils, and eosinophils in BALF. AD particles also increased the production of CysLTs, LTB_4_, and Th2 cytokines (IL-5 and IL-13). However, the CysLT_1_ receptor antagonist (Prl) significantly reduced the numbers of eosinophils and neutrophils in BALF and decreased the production of Th2 cytokines (IL-5 and IL-13), CysLTs, and LTB_4_. These data suggested that AD particles can influence the early stage of antigen-induced immune responses of asthma to an allergen. Furthermore, a CysLT_1_ receptor antagonist may be beneficial to suppress the initial AD-augmented immune response.

Several epidemiological studies have suggested that AD emissions can aggravate asthma, with resulting increases in hospitalization and emergency room visits, deterioration of pulmonary function, and respiratory symptoms [9,10,11,12,13,14,15]. The objectives of these studies were to investigate the association between short-term exposure to heavy AD and asthma, although the effects of long-term exposure to AD on the development of asthma are not clearly understood. The present study showed that co-exposure to AD particles in the antigen sensitization phase of an asthma mouse model was able to increase eosinophilic and neutrophilic airway inflammation. Therefore, long-term exposure to AD may be a risk factor for the development of asthma.

Asthma is a phenotypically heterogeneous chronic disease of the airways, characterized by either predominant eosinophilic or neutrophilic, or even mixed eosinophilic/neutrophilic, inflammatory patterns. Neutrophils play an important role in the pathogenesis of severe asthma [29]. Exposure of patients with asthma to airborne PM can increase their IL-8 concentrations in BALF and blood, as well as IL-8 mRNA expression in bronchial biopsy tissue [30,31]. IL-8 is an important neutrophil chemotaxin in the lower respiratory tract [32], and several studies have demonstrated that neutrophils, but not eosinophils, migrate to the lungs of patients with asthma following PM exposure [33,34]. Therefore, airborne PM can augment neutrophilic airway inflammation in asthma. Similarly, the present study showed that, in the early stage of an antigen-induced immune response, AD particles can increase the number of neutrophils, the concentration of MIP-2/CXCL2 (a murine homologue of IL-8), and the concentration of LTB_4_ in the setting of co-exposure to AD particles and antigen in BALF. LTB_4_ is mainly generated by neutrophils and stimulates neutrophil chemotaxins and activation of the cells [35,36]. As a result, the number and percentage of neutrophils were increased in the mouse airways and lungs. Therefore, exposure to AD during the development asthma may be a risk factor for an increased asthma severity.

CysLT_1_ receptor antagonists are potent and selective antagonists of CysLT activity. However, in the present study, Prl significantly reduced the number of eosinophils and neutrophils in BALF and the concentration of LTB_4_. Previous reports have revealed beneficial therapeutic effects of CysLT_1_ receptor antagonists on neutrophilic inflammatory diseases, including chronic obstructive pulmonary disease, pulmonary fibrosis, and atherosclerosis [37,38,39,40,41]. Shimbori et al. reported that a CysLT_1_ receptor antagonist attenuated LTB_4_ levels in BALF [41]. In addition, Ramires et al. reported that a CysLT_1_ receptor antagonist was able to inhibit 5-lipoxygenase activity and synthesis of LTB_4_ by activated neutrophils [42]. Thus, CysLT_1_ receptor antagonists may have useful effects on neutrophilic airway inflammation augmented by AD particles, as well as on eosinophilic airway inflammation.

GM-CSF is an important chemoattractant for eosinophils and neutrophils, and can potentiate their differentiation and survival [43,44,45]. Th2 cells produce type II cytokines (e.g., IL-5 and IL-13) and eosinophils can produce GM-CSF. Thus, we hypothesize that the decreased neutrophil and LTB_4_ values after Prl treatment may have been dependent on decreased GM-CSF production from Th2 cells and eosinophils, as Prl reduced the number of Th2 cells and eosinophils. However, we did not detect a significant difference in GM-CSF production with and without Prl treatment.

There are several limitations in this study. First, we did not analyze the detailed composition of the AD particles. AD particles mainly consist of mineral dust originating from geological substances, such as silicon dioxide, aluminum oxide, iron (III) oxide, calcium oxide, magnesium oxide [46], anthropogenic metals, and chemicals, although microorganisms that have been introduced by rapid industrial development can also attach to AD particles [47,48]. Thus, the present study was unable to determine which component(s) of AD particles had the strongest effect(s) on augmenting airway inflammation. As noted earlier, urban airborne PM augments asthma, which is primarily dependent on neutrophilic airway inflammation [33,34]. Thus, it is possible that anthropogenic substances attached to AD particles, rather than geological substances, augmented the neutrophilic airway inflammation in the present study. Second, a previous study revealed significant increases in airway hyper-responsiveness (AHR) after intranasal Df administration in the same mouse model [28], although we did not evaluate AHR or pulmonary function in the present study, as we were unable to collect a sufficient amount of AD particles. Third, human patients with asthma are a heterogeneous population, and asthma is affected by unique interactions between genetic and environmental factors. Therefore, the usefulness of Prl as a therapeutic agent to control airway inflammation and bronchoconstriction augmented by AD exposure may vary among individuals.

## 5. Conclusions

The present study demonstrated that co-exposure to AD particles and antigen during the sensitization phase of a mouse model of asthma augmented neutrophilic and eosinophilic airway inflammation, as demonstrated by increased levels of CysLTs and LTB_4_, which could be attenuated by the CysLT_1_ receptor antagonist (Prl).

## Abbreviations

The following abbreviations are used in this manuscript:

AD	Asian dust
BALF	bronchoalveolar lavage fluid
CMC	carboxymethylcellulose sodium
CysLT_1_	type 1 cysteinyl leukotriene
Df	*Dermatophagoides farina*
EIA	enzyme immunoassay
GM-CSF	Granulocyte-macrophage colony stimulating factor
H&E	hematoxylin and eosin
IFN	interferon
IL	interleukin
LT	leukotriene
LTB_4_	Dihydroxy-LT
NS	normal saline
PM	particulate matter
PRL	Pranlukast
SD	standard deviation
